# Point-of-Care Lung Ultrasound Findings in Patients with COVID-19 Pneumonia

**DOI:** 10.4269/ajtmh.20-0280

**Published:** 2020-04-24

**Authors:** Kosuke Yasukawa, Taro Minami

**Affiliations:** 1 Division of Hospital Medicine, Department of Medicine, MedStar Washington Hospital Center, Washington, District of Columbia;; 2 Division of Pulmonary and Sleep Medicine, Care New England Medical Group, Pawtucket, Rhode Island;; 3 Division of Pulmonary, Critical Care, and Sleep Medicine, Department of Medicine, The Warren Alpert Medical School of Brown University, Providence, Rhode Island

## Abstract

Patients with novel coronavirus disease (COVID-19) typically present with bilateral multilobar ground-glass opacification with a peripheral distribution. The utility of point-of-care ultrasound has been suggested, but detailed descriptions of lung ultrasound findings are not available. We evaluated lung ultrasound findings in 10 patients admitted to the internal medicine ward with COVID-19. All of the patients had characteristic glass rockets with or without the Birolleau variant (white lung). Thick irregular pleural lines and confluent B lines were also present in all of the patients. Five of the 10 patients had small subpleural consolidations. Point-of-care lung ultrasound has multiple advantages, including lack of radiation exposure and repeatability. Also, lung ultrasound has been shown to be more sensitive than a chest radiograph in detecting alveolar-interstitial syndrome. The utilization of lung ultrasound may also reduce exposure of healthcare workers to severe acute respiratory syndrome-coronavirus-2 and may mitigate the shortage of personal protective equipment. Further studies are needed to evaluate the utility of lung ultrasound in the diagnosis and management of COVID-19.

## INTRODUCTION

On December 31, 2019, the WHO was alerted to cases of pneumonia with unknown cause in Wuhan, China. The causative agent was later identified on January 7, 2020, as the 2019 novel coronavirus, which was later renamed as severe acute respiratory syndrome-coronavirus-2 (SARS-CoV-2). Since its identification, the number of patients with novel coronavirus disease (COVID-19) continues to increase in the United States and globally.^1^ According to a large cohort study from China, approximately 14% of patients with COVID-19 developed severe illness, and 5% developed a critical disease.^2^ Among hospitalized patients, approximately 20–42% of patients are reported to develop acute respiratory distress syndrome.^3^

It is important to diagnose and to monitor pneumonia in patients with COVID-19. Chest computed tomography (CT) has been shown not only to be more sensitive than reverse transcriptase polymerase chain reaction (RT-PCR) in the diagnosis of COVID-19 but also to correlate with disease progression and recovery.^4–6^ Patients typically have bilateral multilobar ground-glass opacity (GGO) with a peripheral or posterior distribution, and the lesions on CT progress with the greatest severity of radiologic findings visible around day 10 of symptom onset.^7^ Despite its utility, CT is not readily available in many resource-limited settings. In addition, the disinfection of CT machine after the use by a patient under investigation or patients with COVID-19 will result in a delay of care for other patients requiring CT examination. The utility of point-of-care ultrasound (POCUS) has been suggested, but detailed descriptions of lung ultrasound findings are not available.^8–10^ In this study, we evaluated lung ultrasound findings in patients admitted to the internal medicine ward with COVID-19.

## METHOD

This study was a retrospective, observational study. Patients with COVID-19, who were evaluated by lung ultrasound on admission from March 25 to April 7, 2020, were included. Lung ultrasound images of the patients, who received POCUS on admission by the triage/admitting provider (K. Y.) and who were diagnosed with COVID-19 based on the detection of SARS-CoV-2 on RT-PCR from the nasopharyngeal swab, were retrospectively evaluated. In late March, the turnaround time for RT-PCR test for SARS-CoV-2 was approximately 24–48 hours at our institution. During four admitting shifts, an investigator (K. Y.) performed lung ultrasound in one patient with a diagnosis of COVID-19 and 11 patients of 14 patients admitted with possible COVID-19 (three patients had much more likely alternate diagnosis, such as congestive heart failure, based on history, physical examination, and radiographic findings). Among the 11 patients evaluated on lung ultrasound, nine patients were later confirmed to have COVID-19. Stored ultrasound images were carefully reviewed by two investigators (K. Y. and T. M.). We reviewed electronic medical records to determine demographics, comorbidities, laboratory, and radiographic findings. Both investigators have significant training and teaching experience in POCUS. K. Y. has earned the Society of Hospital Medicine/American College of Chest Physicians POCUS Certificate of Completion, completed the Examination of Special Competence in Critical Care Echocardiography of the National Board of Echocadiography (NBE), and is a member of the POCUS committee at his institution. T. M. is a national and international instructor and a director of several courses, and he also directs POCUS training at his own institution and has completed the Examination of Special Competence in Adult Echocardiography by the NBE.

The study was approved by the MedStar Health Research Institute Institutional Review Board (IRB ID: STUDY00002254).

### Point-of-care lung ultrasound examination.

Lung ultrasound was performed using a phased array transducer (Sonosite Edge II, Fujifilm Sonosite, Bothell, WA, with P19 transducer, or Lumify, Philips, Reedsville, PA, with S4-1 transducer). Ultrasound examinations were performed along the midclavicular line in the bilateral anterior chest wall and the scapular line and interscapular regions in the posterior chest wall at the bedside by an experienced physician (K. Y.) while the patients were sitting up. The transducer was covered with a probe cover, and the transducer and tablet/portable ultrasound device were cleaned with disinfectant wipes after each use.

## RESULTS

**Figure 1.**
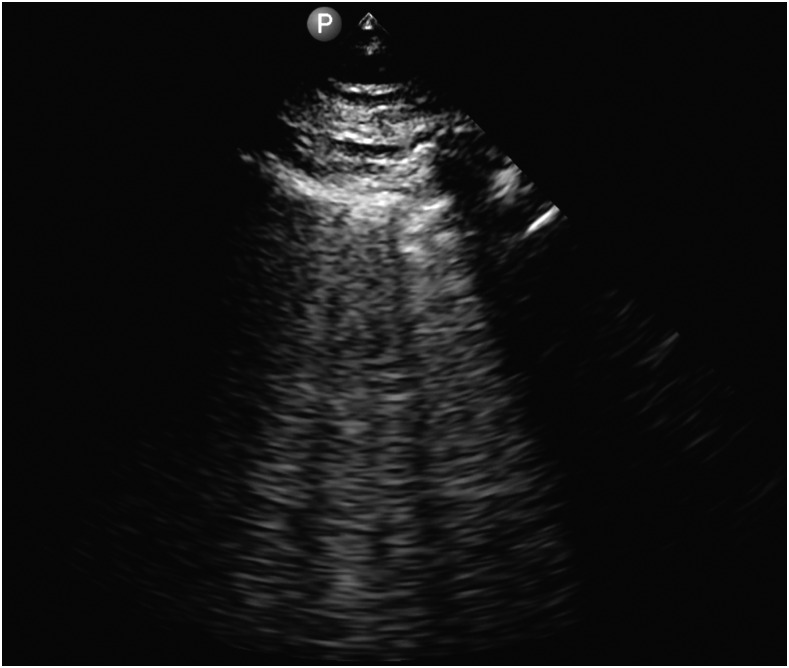
Glass rockets.

**Figure 2.**
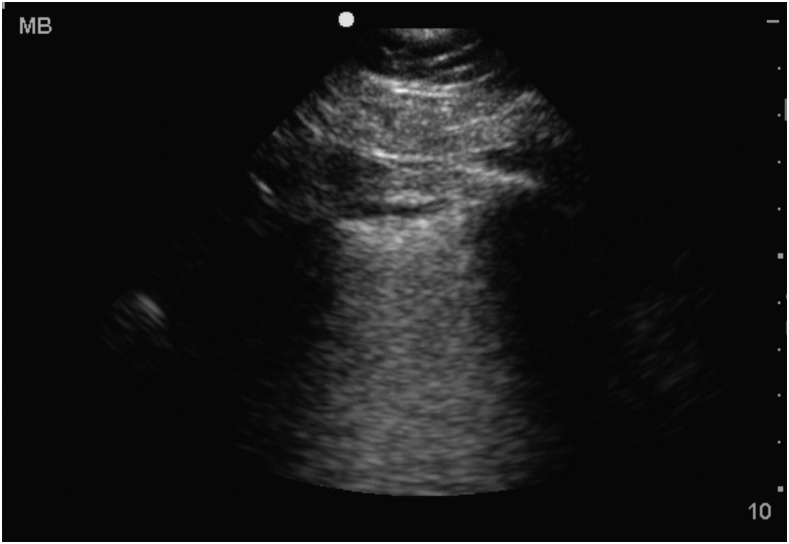
The Birolleau variant (white lung).

**Figure 3.**
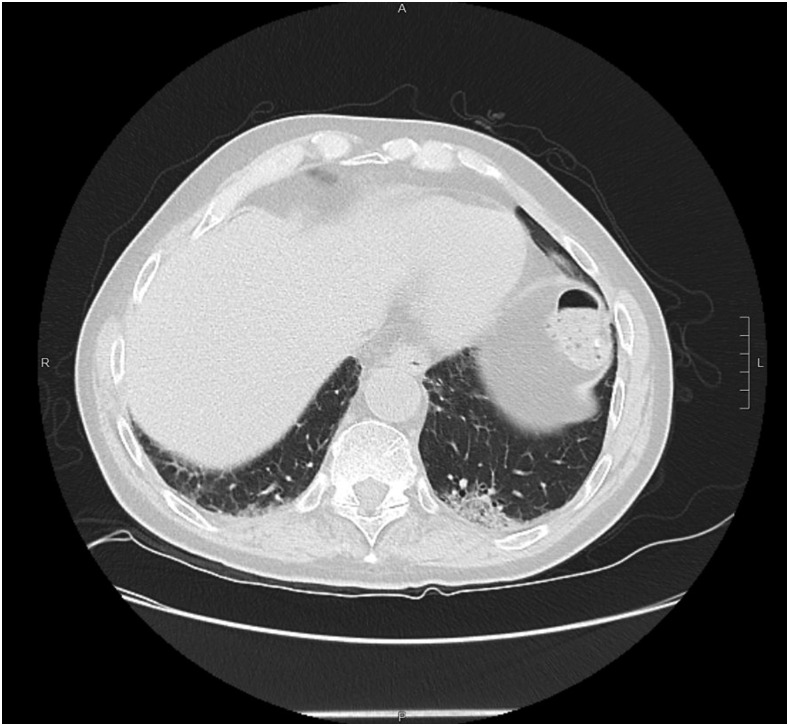
Chest CT showing a mixed density opacity in the left lower lobe.

**Figure 4.**
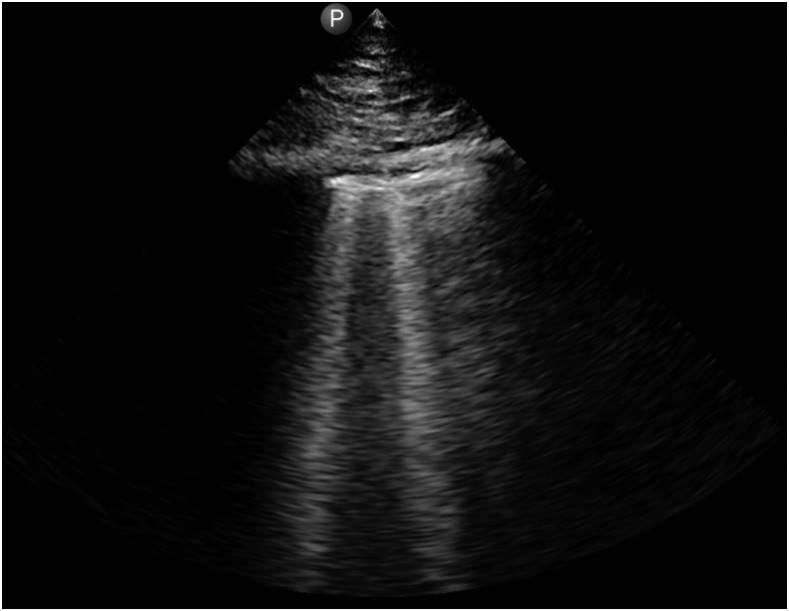
Confluent B lines.

**Figure 5.**
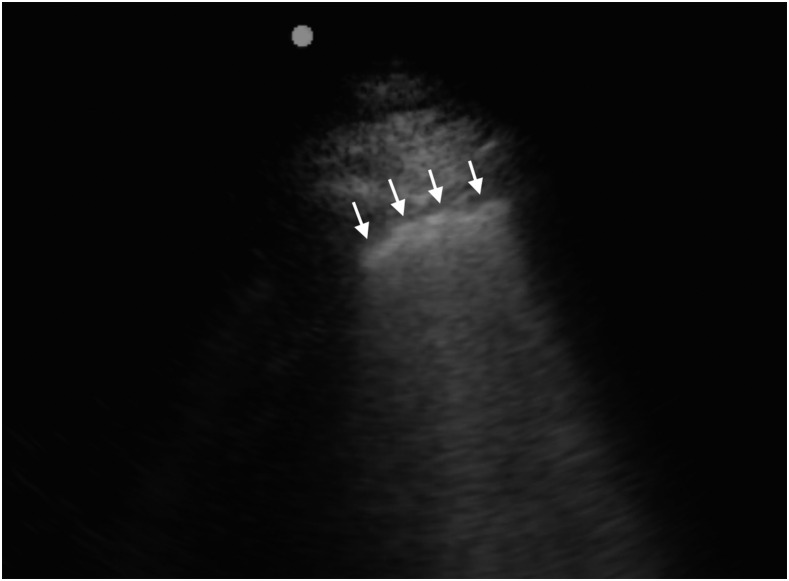
Thick irregular pleural line.

**Figure 6.**
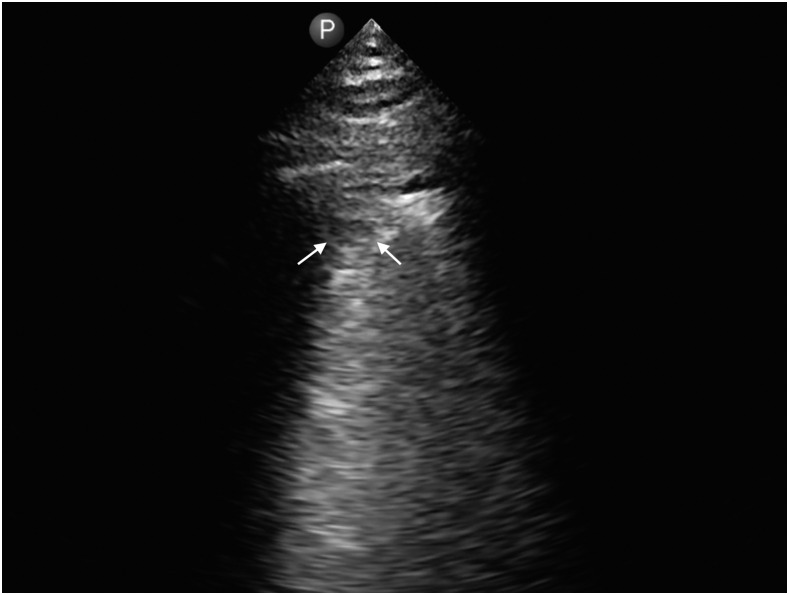
Small subpleural consolidation.

A total of 10 patients were identified. The clinical characteristics of 10 patients are described in Table 1. One patient (patient 3) required transfer to intermediate care unit, and another patient (patient 9) required transfer to intensive care unit. Four of the 10 patients (patient 3, 7, 9, and 10) required administration of oxygen via a non-rebreather mask at a rate of 15 L per minute. Although three patients remain hospitalized at the time of this manuscript writing, none of them have required mechanical ventilation. Abnormal lung ultrasound findings were detected in all of the patients (Table 2). All of the patients had characteristic glass rockets (five B lines or more: Figure 1, Supplemental Videos 1 and 2),^11^ and five of the 10 patients had the Birolleau variant (Figure 2). The Birolleau variant, also called white lung in the literature, is an extreme variant of glass rockets where entire Merlin’s space (the space between the pleural line, the rib shadows, and the lower border of the screen) is hyperechoic.^12^ Two patients had septal rockets (three or four B lines between two ribs). One patient (patient 2) had a chest CT, which showed mixed density opacifications in the peripheral lungs (Figure 3), and the lung ultrasound on the corresponding lesions demonstrated the Birolleau variant. All of the patients had confluent B lines (Figure 4, Supplemental Video 3), and thick, irregular pleural lines (Figure 5, Supplemental Video 4). Small subpleural consolidations (Figure 6, Supplemental Video 5) were detected in five patients, and consolidation was seen in one patient. Pleural effusions were not detected in any of the patients. Both investigators agreed on lung ultrasound findings in most of the stored images. However, when there was a disagreement and a consensus could not be reached even after reviewing the image together (the presence or absence of the Birolleau variant in one patient), the finding was read as negative.

**Table 1:** Demographic and clinical features of study patients

Patient	Age (years)	Gender	Underlying disease(s)	Presenting symptoms	SpO_2_ on admission (room air)	WBC (k/µL) (4.0–10.8)*	LDH (µ/L) (< 246)*	D-dimer (mcg/mL) (< 0.5)*	CRP (mg/L) (< 3)*	ESR (mm/hours) (< 16)*	Ferritin (ng/mL) (< 148)*	NT-proBNP (pg/mL) (< 137)*	Outcome
1	31	Male	None	Fever, cough, and dyspnea	93%	4.8	413	0.81	79.4	73	966.9	Not done	Discharged
2	79	Male	Rheumatoid arthritis	Cough and dyspnea	94%	3.1	344	0.48	55.7	31	274	115	Discharged
3	56	Male	Hypertension and obstructive sleep apnea	Cough, fever, and dyspnea	93%	7	346	0.33	96.1	45	613.6	22	Transferred to the intermediate care unit and remains admitted (required oxygen administration via NRB mask)
4	38	Female	Nonischemic cardiomyopathy (40%), obesity, and asthma	Cough and dyspnea	95%	5.5	211	0.4	68.9	None	125.4	20	Discharged
5	58	Female	Asthma	Cough, fever, and dyspnea	96%	5.9	286	Not done	/38.1	40	210.1	9	Discharged
6	71	Female	Hypertension, hyperlipidemia, and obesity	Cough, fever, and dyspnea	93%	5	Not done	Not done	63.1	> 85	301.1	187	Discharged
7	42	Male	None	Cough, fever, and dyspnea	91%	7	266	< 0.27	> 190	71	608.4	Not done	Discharged (required oxygen administration via NRB mask)
8	58	Male	Hypertension and atrial fibrillation	Cough	96%	3.8	248	Not done	53.9	21	360.7	561	Discharged
9	54	Male	Obesity	Cough, fever, and dyspnea	92%	9.4	509	1.85	171	39	1,096.2	Not done	Transferred to the intensive care unit and remains admitted (required oxygen administration via NRB mask)
10	45	Male	Hyperlipidemia	Cough and dyspnea	89%	6.6	544	1.35	128	> 85	673.4	< 5	Remains admitted (required oxygen administration via NRB mask)

The criteria to rule out heart failure with NT-proBNP vary with gender, age, and renal function.

* Normal range.

**Table 2:** Findings on lung ultrasound

Patient	Chest X-ray report	Findings on lung ultrasound						
		Septal rockets	Glass rockets	Birolleau variant	Confluent B lines	Thick, irregular pleural lines	Small subpleural consolidation	Consolidation
1	Bilateral multifocal airspace opacities	+	+	+	+	+	+	−
2	Faint opacity in the right upper lobe, and bibasilar opacities	−	+	+	+	+	+	−
3	Bilateral faint airspace disease	−	+	−	+	+	−	−
4	Multifocal airspace disease particularly in the right mid and lower lung	−	+	+	+	+	+	−
5	Mild pulmonary vascular venous congestion	−	+	−	+	+	+	−
6	Patchy airspace disease in both lung bases	−	+	+	+	+	−	−
7	Multifocal patchy airspace disease	−	+	−	+	+	+	+
8	Patchy irregular opacities in the lower lobes	+	+	−	+	+	−	−
9	Bilateral scattered airspace opacities	−	+	−	+	+	−	−
10	Bilateral peripheral patchy airspace opacities	−	+	+	+	+	−	−

## DISCUSSION

We found that characteristic glass rockets with or without the Birolleau variant were present in all of the 10 patients with COVID-19 admitted to the general medicine floor. Glass rockets and the Birolleau variant indicate ground-glass areas on CT and in turn a high-degree interstitial syndrome.^12^ Although glass rockets and the Birolleau variant can be seen in patients with cardiogenic pulmonary edema, none of our patients was diagnosed with decompensated congestive heart failure. One patient with a history of atrial fibrillation (patient 8) had elevated NT-proBNP, but the chest radiograph was not consistent with pulmonary edema, and the patient improved without administration of diuretics. One patient’s (patient 5) chest radiograph reported pulmonary venous congestion, but the patient’s NT-proBNP was low (9 pg/mL), and the limited cardiac ultrasound at bedside showed hyperdynamic left ventricular function, suggesting hypovolemia.

Of note, all of the patients had confluent B lines in our study. Small subpleural consolidations were observed in five patients. Lung ultrasound studies on influenza pneumonia reported subpleural consolidations and confluent B lines aided in distinguishing between viral pneumonia and bacterial pneumonia.^13,14^ In our study, thick, irregular pleural lines were also present in all patients. In the setting of multiple B lines, the presence of irregular, thick pleural lines suggests inflammatory process of the pleura rather than cardiogenic pulmonary edema.

Studies on ultrasound findings in patients with COVID-19 are limited, but our findings are consistent with prior reports of the presence of “diffuse B-line pattern,” thick irregular pleural lines, confluent B lines, and subpleural consolidation. Large consolidations were only observed in one patient in our study, but the presence of consolidation likely correlates with disease progression based on prior studies on CT findings in patients with COVID-19. As most patients with COVID-19 develop GGOs in the peripheral distribution which progress over time to form more consolidative changes, the ultrasound can likely detect most symptomatic patients with COVID-19 who require hospitalization. The glass rockets, confluent B lines, thick irregular pleural lines, and subpleural consolidations are likely not specific to COVID-19 and can be observed in other conditions such as other viral pneumonias and ARDS. However, these findings, particularly when combined, can be an aid for diagnosis during the COVID-19 pandemic when pretest probability is high.

Lung ultrasound has multiple advantages over chest radiograph and chest CT in the diagnosis and management of patients with COVID-19. Lung ultrasound can be easily repeated at bedside without exposing patients to radiation. Also, lung ultrasound has been shown to be more sensitive than chest radiograph in the diagnosis of alveolar-interstitial syndrome.^15^ Lung ultrasound can likely detect lung lesions earlier than chest radiograph when the lesions are located adjacent to the pleura. Furthermore, using POCUS instead of chest radiograph and chest CT can reduce exposure of SARS-CoV-2 to healthcare workers, such as transport staff and radiologic technicians, which may also help mitigate personal protective equipment shortages experienced in many healthcare facilities.^16^

Our study has multiple limitations. This is a retrospective study with a limited number of patients. Future studies with a larger number of patients are needed to better evaluate the lung ultrasound findings in patients with COVID-19 and to evaluate the utility of lung ultrasound in the management of patients with COVID-19. More detailed evaluation with scoring system, such as used in critical care,^17,18^ may provide prognostic information in patients admitted with COVID-19. Image acquisition was performed by only one expert sonographer; thus, these findings may not be obtained by novice sonographers. Also of note, this study was conducted in patients who required admission to general internal medicine service. Further studies are needed to characterize ultrasonographic findings of patients with COVID-19 in other clinical settings.

In conclusion, this small preliminary study suggests that glass rockets with or without the Birolleau variant, confluent B lines, thick irregular pleural lines, and subpleural consolidations are typical lung ultrasound findings in patients with COVID-19 pneumonia. The presence of these findings is helpful when evaluating patients with suspected COVID-19. In resource-limited settings where chest radiograph, CT, and RT-PCR are not readily available or turnaround time is long, lung ultrasound can be an aid in the diagnosis of COVID-19.

## Supplemental videos

Supplemental materials
